# Innovative Chorioallantoic Membrane Model as Valuable Tool in Diagnostics and Testing of Domestic Animal Cancers

**DOI:** 10.1155/tbed/1876572

**Published:** 2026-01-03

**Authors:** Anna Sczasny, Jerzy Gubernator, Anna Jaromin

**Affiliations:** ^1^ Center for Translational Research and Molecular Biology of Cancer, Maria Skłodowska-Curie National Research Institute of Oncology, 44-102, Gliwice, Poland, onkologia.krakow.pl; ^2^ Department of Lipids and Liposomes, Faculty of Biotechnology, University of Wrocław, 50-383, Wrocław, Poland, uni.wroc.pl

**Keywords:** animal cancers, CAM, chicken embryos, chorioallantoic membrane, in ovo/ex ovo model

## Abstract

In recent years, particular attention has been paid to the possible connections, similarities, and potential uses of animals, especially pets (dogs and cats), in research on the causes, characteristics, and treatment of cancers occurring in pets and humans. One of the most promising experimental research models used to explore these issues is the avian embryo chorioallantoic membrane (CAM). This review aims to highlight the problem of the occurrence of cancers in domestic animals, placing emphasis on types, incidence, and predispositions of dogs and cats. Methodology and applications in cancer studies of this unique model were presented in detail. Moreover, the advantages and disadvantages of this diagnostic tool, as well as potential and future perspectives, were also described. This review confirms that cancer research can be conducted without the use of animals. Furthermore, the CAM can provide a robust and reliable model for this type of research and provide translational potential as an ethical, cost‐effective model bridging laboratory and clinical research.

## 1. Introduction

The first use of chorioallantoic membrane (CAM) dates back to 1911 [1], when two scientists demonstrated chicken sarcoma tumor transplantation on CAM, thus over a century ago. Since then, the advantages of the model and its desired features have been gaining in value. In the last century, it has been used quite intensively for many studies related to angiogenesis, due to its features. In addition to tumors, viruses and bacteria have also been successfully investigated [2, 3]. Furthermore, it has found applications in broadly understood toxicological studies (nanotoxicity, skin, and ocular) [4], as well as in tissue engineering and biomaterials research [5]. Most research on the CAM model is devoted to studies on the activity and toxicity of various anticancer drugs. Some research also concerns skin models and structure [6, 7]. Although it was once overshadowed by new techniques, the CAM model is now experiencing a major revival.

The CAM model is used where other methods have their limitations, both technical and ethical [8]. As in many countries, approval by an ethics committee is not required up to a certain point. Regardless of the bird species, all scientific activities must be completed before the embryo begins to feel pain; otherwise, permission is required. Nevertheless, it perfectly fulfills the “3R” principle. This principle was established almost 70 years ago by William Russell and Rex Burch to regulate issues related to conducting research on animals [1]. “3R” refers to three words, namely replacement, reduction, and refinement. According to these, the goal of scientists is to design the experiment in such a way as to replace animals, for example, with computer analysis and reduce their number to a minimum, maximizing the amount of data obtained from one individual. And ultimately also to take care of their well‐being and provide conditions without pain and suffering [1, 9]. The CAM, once used mainly in angiogenic, oncological, dental, and xenograft research, is currently also used in microbiology for studying invasiveness and interaction of various bacterial strains, in tissue engineering, thus giving promising results in evaluating growth factors or the material used itself. Moreover, it allows us to explore hemodynamics or trafficking of immune cells and even gives the prospect of using it in investigating the early stages of development of the human placenta by studying one of the placental cell types—trophoblasts [8, 10, 11].

## 2. CAM Model

### 2.1. Chicken Embryo Anatomy and CAM Development

**Figure 1:**
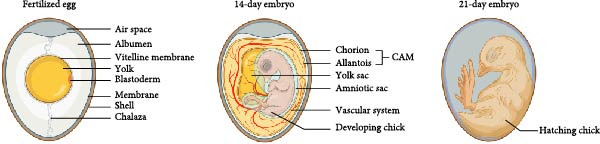
Structure of chicken egg during embryonic development (created with BioRender).

To understand what the CAM is and what its functions are, it is necessary to examine the anatomy of the avian embryo during its development. Figure 1 provides a clear illustration of the progressive changes occurring inside the egg during this process.

One of the key structures in early development is the blastoderm (embryonic disc), located on the surface of the yolk. This is particularly important for the formation of the CAM. Therefore, it is important to maintain the integrity of the yolk during the initial stages of incubation. For yolk protection, densified and twisted protein strings called chalaza are responsible. The development process of the chicken embryo is highly dynamic, and the visible signs of life appear in less than 24 h since the start of incubation. Initially, single‐layer clusters of ecto‐ and endoderm cells become visible on the embryonic disc, which will later differentiate into the skin, digestive system, nervous system, and extraembryonic membranes, including CAM. By the end of the first day, the mesoderm responsible for the formation of the heart, circulatory system, and skeletal system also develops. Therefore, on the second day, the enlarging area of the embryonic disc, its vascularization, beating heart, and slowly outlined shapes of the microscopic‐sized embryo can be observed [1]. The CAM arises from the fusion of two out of four extraembryonic membranes that are found to be key protectors and nutrient suppliers during embryonic development. The allantois emerges on day 3, but it develops rapidly from days 4 to 10 [1]. Initially, it is a small, thick‐walled pocket of endodermal origin directly extending from the ventral part of the hindgut. Its function is to store metabolic waste, mainly urea and uric acid. In contrast, the chorion, unlike the allantois, has differentiated from the ectoderm and lines the inner surface of the eggshell, allowing gases to pass through and gas exchange between the embryo and the external environment. According to various sources, these last two membranes merge between days 4 and 5, even 6, of incubation, forming the so‐called CAM. Their merger is facilitated by the mesodermal layer. The CAM progressively grows until it surrounds the contents of the egg around day 10, while by day 12 it completely covers the inside of the shell, and by day 13 the membrane is fully differentiated [1, 11, 12]. The CAM is not unique to birds, but it is a common feature of amniotes, including mammals and reptiles. Despite its simple structure, it is a highly vascularized, lymphatic network‐bearing extraembryonic transparent membrane that, in addition to respiratory gas exchange, also allows for the transport of calcium, acid–base homeostasis, and ion reabsorption from amniotic fluid [12]. The vascularization develops in the allantoic mesoderm, consisting of two arteries and one vein, constituting a direct connection with the embryo. As development progresses, these vessels branch out into the chorion, providing access to ambient air. As with the amnion and amniotic fluid, the CAM membrane gradually regresses with the increasing independence of the embryo and finally disappears before hatching [11].

### 2.2. CAM Cultivation Approaches

**Figure 2:**
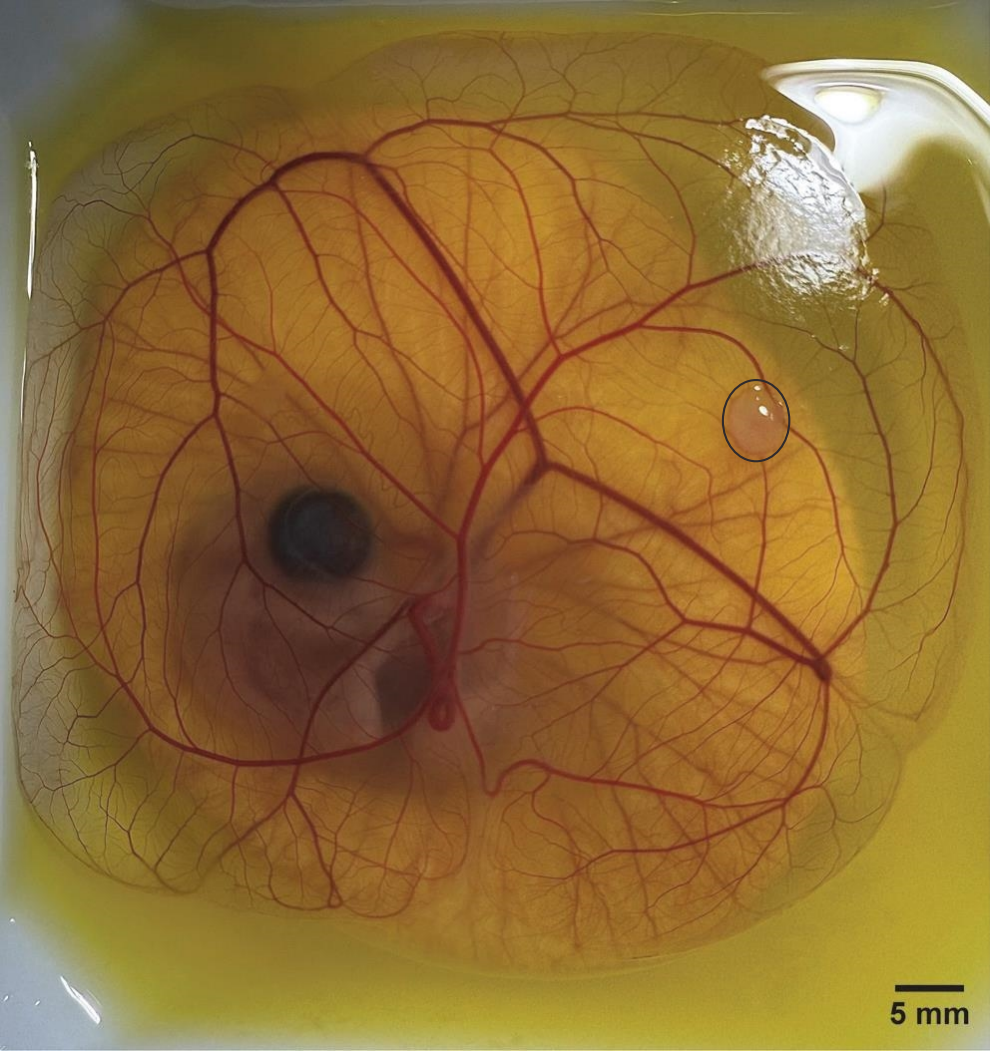
Ex ovo cultivated chorioallantoic membrane (CAM) of a chicken embryo on day 10 of incubation, maintained on a polystyrene weighing boat. A tumor developing from the BxPC‐3 cell line is marked with a black circle (image provided by authors).

So far, embryo cultures have been carried out either using the traditional, noninvasive method, which most closely mimics natural conditions, that is in the shell, the so‐called in ovo, or the less conventional but providing much greater research possibilities, that is outside the egg, the so‐called ex ovo, preferably in a transparent container. Both of these approaches have their advantages and disadvantages. In both methods, favorable, desired growth conditions are equally important—temperature, which in most available sources was equal to 37°C, humidity ranging between 60% and 70% with 60% most often chosen, rotation, ventilation, which can affect metabolism, transformations, overall the functioning of the developing organism, as well as CAM performance It should be noted, however, that in the case of culture outside the eggshell, another disturbing factor is introduced—the container and transferring the contents of the egg. For both approaches, the procedure begins with a 3‐day incubation of fertilized eggs with rotation, to ensure proper development of the CAM. Turning eggs during incubation prevents undesirable embryo positions and, thus, attachment of the emergent CAM membrane to the shell. In the case of in ovo, the shell is gently opened, and the holes are secured with semi‐permeable films, which affects the overall sterility of the entire procedure and changes in oxygen tension. In the case of ex ovo culture, the 3‐day embryo with the remaining content must be transferred to a container. This is early enough that all the membranes are just developing and are not yet attached to the shell, and late enough that the embryo has a better chance of surviving the disturbances associated with it than in the first stage. Kundeková et al. [1] and Marshall et al. [11] stated that the in ovo method is repeatable, less sensitive to changes, and has a high survival rate, while in the ex ovo method, two scenarios are possible—over 80% survival with an experienced operator and 25% for amateurs. Additionally, a cost‐effective alternative version of the ex ovo culture procedure has been developed, the so‐called cup‐CAM. According to Naik et al. [13], such an approach provides viability at the level of 85%–95%, which is identical to the in ovo method. Regardless of the version of the ex ovo procedure, it has great research possibilities, that is, visualization of the membrane itself is possible already at the stage of day 3, and in the entire volume, it is not limited by the size of the vision window cut in the eggshell (Figure 2).

Additionally, numerous materials can be applied to CAM at once, where again it is not possible in in ovo.

## 3. Cancers in Domestic Animals—Types, Incidence, and Predispositions

Currently, one of the most frequently discussed topics in publications is the problem of the increasing incidence and mortality of cancer in humans. According to data from the GLOBOCAN report conducted for the International Agency for Research on Cancer (IARC) [14], in 2022 alone, 20 million people were affected by cancer, and forecasts by 2050 predict a 77% increase in this number. Amidst all this focus on diseases affecting humans, those that are increasingly common, yet rarely detected and studied in animals, have been overlooked. The use of pets in experiments is controversial and not entirely ethical due to the potential pain and suffering, and the bond between humans and pets [15], while for others, it remains acceptable. A shared environment, and therefore cancer‐causing factors [16], is becoming the basis for research in comparative oncology. This field relies on identifying similarities and differences between naturally occurring cancers in animals, primarily cats and dogs, with the goal of providing plausible methods of treating humans. However, it should be noted that although pets share environments and habits with humans, humans do not undergo genetic manipulation, controlled sterilization, or reproductive restriction as is common in laboratory animals. Moreover, it is important to clarify that domestic animals themselves are not used in experimental research. Studies involve animal species that may also be kept as pets, but which originate from licensed facilities and are handled under appropriate ethical approval. According to the European Animal Research Association (EARA) [17], regarding research on animal models, dogs are used as a last resort when other well‐established models prove insufficient. However, considering their well‐being and the sympathy bestowed upon them, it is worth considering whether other models, such as rodents (mice, rats) or rabbits, which remain the standard laboratory animals, are truly incapable of meeting the challenges posed.

### 3.1. Canine Neoplasms

Cancer is the leading cause of death in dogs, accounting for as much as 27% of all deaths. Although statistical data appear limited, according to [18, 19], a study of the incidence of tumors in dogs in Switzerland conducted between 1955 and 2008 and between 2008 and 2020 revealed that tumors were detected in over 51% and 45% of examined individuals, respectively. Of these, 47% and ~32%, respectively, were classified as malignant tumors.

The most common types include: adenomas and adenocarcinomas, fibromas and fibrosarcomas, hemangioma and haemangiosarcomas, lymphomas, osteomas and osteosarcomas, mast cell tumors, squamous cell carcinomas, and skin cancers. The distribution of individual cancer types has changed. Adenoma/adenocarcinoma was unmatched in the earlier study, affecting as much as 18% [20]. Cancers were most frequently detected in medium‐ and large‐sized dog breeds. According to another source, the body size or weight of the animal is a factor increasing the risk of the disease [21]. Age is a typical factor for each of the above‐mentioned cancer types. A detailed breakdown of tumor types, incidence, and associated risk factors such as breed, age, and neutering status is provided in Supporting Information 1: Table S1.

### 3.2. Feline Neoplasms

The most common causes of death in cats include urinary tract diseases, circulatory system diseases, and cancer. However, unlike in dogs, cancer did not rank highest. According to Inoue and Sugiura [22] in a study conducted in Japan, cancer was the second most common cause after urinary tract diseases, as reported in. Data availability regarding this condition is even more limited than for dogs. Again, based on data and observations described in [23], it is concluded that in a group of feline patients studied between 1965 and 2008, nearly 35% were diagnosed with cancer, 80% of which were malignant. The list of most common cancers in cats is much shorter than in dogs, but the types of cancers diagnosed have not changed. These include adenoma/adenocarcinoma, fibroma/fibrosarcoma, lymphoma, squamous cell carcinoma, and osteoma/osteosarcoma. The values obtained were significantly higher compared to both studies conducted on canine patients in Switzerland, and were even similar, with no significant differences except for the extremely rare bone tumor (osteoma/osteosarcoma), whose incidence is estimated at less than 5 per 100,000 cases [24]. Some cat breeds were associated with specific cancers. However, more often, a specific breed was a protective factor—it reduced the risk of the disease. Due to the limited availability of recent data on feline cancer, the most comprehensive findings remain based on retrospective studies, and a detailed summary is provided in Supporting Information 2: Table S2.

### 3.3. Others

Although there exist studies on the incidence of cancer in other domestic or companion species (e.g., rabbits, rodents, certain reptiles, and ornamental birds) [25–29], and the types of cancers found in them overlap with those reported for dogs and cats, to date, no studies have been conducted on the CAM model. Therefore, although cancer has been documented across a wide range of domestic animals, the CAM model‐related literature currently covers only dogs and cats.

## 4. CAM as Valuable Tool in Diagnostics and Testing of Domestic Animal Cancers

In publications on human cancers, the CAM model predominates. Unfortunately, the number of available publications on animal cancer involving CAM is very limited. Since human cancers dominate most CAM research, making it difficult to obtain sufficient information on animal tumor cultures. We might ask whether this is due to the fact that the CAM method is not popular in pets’ cancer research, or whether it simply does not provide the same information as human cancer models.

### 4.1. Methodology

It has been reported [30–32] that the CAM model is widely used in human medicine and preclinical oncology research. Its advantages are described in terms of observing processes and changes occurring in developing cancers, including angiogenesis, metastasis, and the effectiveness of administered drugs. At the same time, the limited research on diseases affecting animals is mentioned, emphasizing its potential use in veterinary medicine, particularly oncology. To date, the model has been used in veterinary oncology research to a limited extent with respect to tumor types. However, the model’s plasticity and adaptability to modifications allow for the expansion of research and, to some extent, approaching that conducted in humans. Currently, most publications are devoted to studies on human cancers, which have been shown to be based on the classic, more natural in ovo (in‐shell) model [33]. However, when collecting all recently published articles studying pets’ neoplasms, no significant predominance of any of the approaches was found (Table 1).

**Table 1:** Overview of approaches in the investigation of animal cancers tested on CAM.

CAM model	Cell line	Cell cultivation conditions	CAM cultivation conditions	Application way	Tumor development	Examination way	References
In ovo	FVAF1	DMEM + 10% FBS, penicillin‐streptomycin (50 IU/mL), fungizone (2.5 mg/mL); at 5% CO_2_, 95% humidity, and 37°C	65% humidity, 5% CO_2_, and 37.5°C with turning at the initial stages	Membrane surface, at day 7, 5 × 10^6^ cells per egg into silicone rings	From day 7 to day 18 (11 days)	H&E and IHS stainings	[30]
In ovo	D‐17	EMEM + FBS, penicillin‐streptomycin (50 IU/mL), amphotericin B (2.5 mg/mL); at 5% CO_2_, 95% humidity, and 37°C	55% humidity, 37°C, with turning at the initial stages	Membrane surface, at day 6, 5 × 10^6^ cells per egg into silicone rings	From day 6 to day 19 (13 days)	H&E staining	[31]
Ex ovo	OSCA‐8 and OSCA‐32	DMEM + 10% FBS, 10 mM HEPES, Primocin (100 µg/mL); at 5% CO_2_, 95% humidity, and 37°C	70% humidity, 39°C in polystyrene weighing boats	Intravenously, at day 11, 1 × 10^5^ cells per egg	From day 11 to day 12 (1 day)	Fluorescent microscopy imaging	[32]
Ex ovo	17CM98, D17, K9STS and HAS	RPMI 1640 + 10% FBS, 1% penicillin/streptomycin, 1% HEPES, 1% sodium pyruvate, 1% NEAA, 1% GlutaMax; at 5% CO_2_, 95% humidity, and 37°C	60%–62% humidity, 37–37.5°C	Membrane surface, at day 9, 4–6 × 10^6^ cells per egg embedded in Matrigel	From day 9 to day 14.5 (5.5 days)	H&E staining	[34]
In ovo	FFS1 and FFS3	DMEM + 10% FBS, 4500 mg/L glucose, penicillin‐streptomycin (50 IU/mL), amphotericin B (2.5 mg/mL); at 5% CO_2_, 95% humidity, and 37°C	65% humidity, 5% CO_2_, and 37.5°C with turning at the initial stages	Membrane surface, at day 6, 5 × 10^6^ cells per egg into silicone rings	From day 6 to day 19 (13 days)	H&E and IHS stainings	[35]
In vitro/ex ovo	FMC	DMEM + 10% FBS, penicillin‐streptomycin (100 µg/mL); at 37°C	60% humidity, 5% CO_2_, and 37.5°C with turning at the initial stages	Membrane surface, at day 7, 1 × 10^5^ cells per egg embedded in Matrigel	From day 7 to day 17 (10 days)	H&E, cell sorting, and western blot	[36]

*Note: “*in ovo, in vitro, and ex ovo” refer to in shell, in glass, and shell‐less CAM cultivation, respectively; “FVAF1” stands for feline vaccine‐associated fibrosarcoma cell line; “FMC” stands for feline mammary carcinoma cell line; “FFS1 and FFS3” stands for two feline fibrosarcoma cell lines; “D‐17” stands for canine adherent osteosarcoma cell line; “17CM98, K9STS, and HAS” stands for canine oral melanoma, soft tissue sarcoma, and hemangiosarcoma, respectively; “OSCA‐8 and OSCA‐32” stands for two canine osteosarcoma cell lines.

All methods, although differing in minor aspects such as lower or higher humidity, temperature, or the presence or absence of egg rotation during the initial stages of embryonic development, are derived from well‐described protocols used in human cancer research. These minor differences in culture conditions may simply result from the devices used, the model itself, whether the embryo develops in or out of the shell, as well as the available equipment and its technical advancement.

Regarding inoculation and initiation of tumorigenesis, minor differences may also result from operator preferences, the characteristics of the study being conducted, or the cells themselves. Ademi et al. [34] discovered that in the case of human cancer cell lines, there was no need to use an external extracellular matrix (ECM) such as Matrigel, but for other animal‐derived cell lines, it was necessary to ensure proper cell engraftment. The route of cell administration depended on the purpose of the study. In the study conducted by Małek et al. [32], the goal was not simply to grow cancer on CAM and determine whether a given cell line could grow, but rather to test the extravasation process and the effect of the administered substances. Therefore, administration was intravenous and not, as in the other cases considered, to the membrane surface. The number of cells chosen to initiate tumorigenesis varied slightly between publications. The determination of the appropriate number of cells was based either on serial studies optimizing this number or on studies on human cell lines [35], or on observations of tumor growth from individual lines, often dependent on the characteristics of a given line (its aggressiveness) [34].

The date of initiation of tumor culture on CAM and its dynamics varied between publications. The earliest cells were plated on the membrane was day 6 of embryonic development, and the latest was on day 11. Again, this is due to the purpose of the experiment described in [32] and the characteristics of the cell line—its ability to initiate the process, its dynamics of development, and its malignancy. However, this is not the only reason, as research conducted on animal models, such as mice, rats, or larger organisms, is subject to legal regulations to ensure their well‐being and prevent suffering. The same applies to the chicken embryo model. According to European law, specifically Directive 2010/63/EU of the European Parliament and of the Council of 22 September 2010 on the protection of animals used for scientific purposes [30], the bioethical commission’s approval is not necessary as long as the experiments are completed before hatching. However, according to Swiss legislation, namely Animal Protection Ordinance Article 112 [34], experiments are to end on the day the embryo begins to feel pain, that is, day 14 of development. The time allotted for experiments may also vary between countries where they are performed. Thus, most CAM‐based experiments can be performed without animal ethics committee approval, provided they are terminated before day 14 of incubation. The success of the experiments, that is, the evaluation of the presence and characteristics of the obtained tumors, in most publications was based on histopathological analysis using hematoxylin and eosin (H&E) staining and immunohistochemical analysis based on a specific marker characteristic for a given tumor type (Ki‐67 in [35] or CD133+ in [36]). This, as well as other factors (presence of necrosis, mitotic index, angiogenesis), were used to determine similarities and differences with the characteristic features of tumor tissues of specific types. This determined whether a tumor that met the desired criteria had been successfully cultured. However, as with other aspects of the methodology, here too, somewhat more extensive, complex analyses were performed, utilizing other techniques, including cell sorting (flow cytometry), protein analysis, and fluorescence microscopy. These methods were used to address the specific nature of the study, that is, to exclude or confirm the presence of a specific cell type and/or to select specific ones for further steps, or to visualize the location of cells within the tissues of the developing embryo [32, 36].

### 4.2. Scope of Research Conducted and Main Outcomes

Surprisingly, despite the scarcity of publications on veterinary oncology research conducted on CAM, they provide quite extensive analyses and promising results. Some publications focused solely on establishing the possibility of observing the tumorigenesis process on the CAM membrane and demonstrating similarities or differences with a specific type of cancer [30, 31, 35]. Such studies were an integral part of almost all of the publications considered, and in all of them, they met the established criteria, which underscores the model’s ability to mimic the original conditions of tumorigenesis. However, there were also publications where, in addition to the effective method of tumor cultivation, cell susceptibility to well‐known therapies was tested, both traditional (radiation and chemotherapy) [36] and more modern ones, such as anti‐angiogenic immunotherapy [34] or therapy based on the use of nanocarriers [32]. An overview of theconducted researches and main findings is presented in Table 2.

**Table 2:** Key aspects of considered publications.

Study objective	Key experimental steps	Research findings	References
Isolation and cultivation of feline vaccine‐associated fibrosarcoma cell line on CAM	– FVAF cell line isolation from patient‐derived tumor‐growth of inoculated cells on CAM, and tumor development – Histopathological and immunohistochemical evaluation of developed tumors	– General characteristics (size, shape, presence of blood vessels) of developed were determined tumors – Histopathological and immunohistochemical confirmation of grown tumors origin	[30]
Determination of the ability of commercially derived canine adherent osteosarcoma cell line to form solid tumors on CAM	– Tumorigenesis of inoculated cells on CAM – Histopathological and immunohistochemical evaluation of developed tumors	– General characteristics (size, shape) of developed tumors were determined – Histopathological and immunohistochemical confirmation of grown tumors origin – Specific features of osteosarcoma (necrosis and hemorrhage) were observed	[31]
Cultivation and characterization of two feline fibrosarcoma cell lines (intermediate to high malignancy) on CAM	– Growth kinetics assessment – Tumorigenesis of inoculated cells on CAM – Histopathological and immunohistochemical evaluation of developed tumors	– General characteristics (size, shape, consistency, and presence of blood vessels) of developed tumors were determined – Histopathological and immunohistochemical confirmation of grown tumors origin	[35]
Characterization of feline mammary cancer stem cells in terms of expression of specific stem cell markers, invasiveness, and treatment (radiation and chemotherapy) resistance	– In vitro assays such as: invasion assay, tumorigenicity, radiation, and cytotoxic drugs sensitivity – In vivo assays such as: tumorigenicity in mice, FMC stem cells migration potential on CAM – Western blot and cell sorting	– Tumor initiation by feline stem cells in both mice and CAM was assessed – High FMC stem cells invasion and treatment resistance marker‐dependent (CD133^+^) potential were determined	[36]
Evaluation of the gold nanoparticles inhibitory potential on canine osteosarcoma cells of different aggressiveness	– Gold nanoparticles (AuNP) cytotoxicity test – Assessment of AuNP cellular uptake, accumulation, and their antiextravasation activity – Assessment of osteosarcoma cells extravasation delivered intravenously on CAM	– Localization of fluorescently labeled osteosarcoma cells and assessment of their extravasation – Confirmation of the inhibitory effect of AuNP on extravasating canine osteosarcoma cells	[32]
Determination of anti‐angiogenic and hypoxia‐inducing capabilities of known and available inhibitors (e.g., Avastin)	– Tumorigenesis of inoculated cells on CAM – Assessment of tumor cell metastasis – Quantification of tumor growth and perfusion − Antiangiogenic treatment	− Tumor‐forming cell lines were determined − Positive effects of anti‐angiogenic treatment were recorded − Blood perfusion was visualized and quantified − Metastasis of developing tumors in response to anti‐angiogenic treatment was quantified	[34]

To begin with, the more basic ones, which focus on the process of tumorigenesis and characterization of the obtained tumors. The observations made (visibility of neoplasm on CAM, the angiogenesis process, shape, consistency, and size) and the H&E staining methods, as well as those based on specific antibodies used, allowed, first, to determine the presence of cancer cells in the cultivated neoplasms. Second, authors precisely determined their shape, structure, type, and division dynamics, thus their malignancy. By using the CAM, in addition to culturing a tumor from material collected from the patient without exposing them to stress and pain, and conducting a histopathological examination, it is also possible to observe the developing lesion in a living organism. Although the literature on the use of pets’ cancer cells is currently limited, this approach has been successfully demonstrated in studies conducted on human tumor grafts, including blastomas, pancreatic, colorectal, and breast cancers [37]. Compared to studies conducted on cell cultures, the CAM model provides a broader understanding of the entire process of tumorigenesis, angiogenesis, and metastasis occurring within the body. This may not only be crucial for diagnosis but also for planning further steps in a relatively short time.

As it was demonstrated by Zabielska et al. [30], it was possible to culture and observe feline vaccine‐associated fibrosarcoma from cells isolated from tissues collected from the patient (FVAF1). Furthermore, they confirmed their consistency with the histopathological picture characteristic of vaccine‐associated feline sarcoma (VAFS), thanks to staining that allowed for the identification of specific proteins such as vimentin, desmin, or cytokeratin. By analyzing the shape, nucleus, presence of necrotic areas and blood vessels, and mitotic rate, they were able to classify the obtained neoplasms in terms of their malignancy. A similar analysis was conducted by Zabielska‐Koczywąs et al. [35], where not one but two feline fibrosarcoma cell lines with varying degrees of malignancy were considered (FFS1 and FFS3). Again, it was possible to determine the similarity of the cultured tumors to the characteristics of spontaneously occurring feline fibrosarcomas, using previously developed criteria (mitotic rate, degree of necrosis, and degree of differentiation). However, to confirm whether the tumors cultured from each cell line corresponded to its original grade of malignancy, the immunoreactivity of both tissues for two proliferation markers, Ki‐67 and PCNA, was tested. Both markers are considered good predictors of response to chemotherapy in some human and pet cancers. Based on the obtained values, both cell lines were classified according to the original assumptions, with FFS1 representing grade 3, meaning significantly altered histopathologically with high malignancy, and FFS3 with locally altered histopathology indicative of intermediate malignancy.

An identical evaluation of tumor culturing capacity was conducted by Walewska et al. [31], with the difference that the cell line used was commercial and a canine adherent osteosarcoma, another type of cancer most commonly found in domestic animals. In addition to the previously described successes, here authors also observed typical processes occurring in the development of osteosarcoma, that is, angiogenesis, necrosis, and above all, hemorrhage, which again highlights the potential offered by the CAM model.

Considering the publications, where the basic analysis was expanded to include testing for response to various therapies, it is important to begin with [36], the only one from researches presented in Table 2 to conduct studies using feline mammary carcinoma (FMC). Furthermore, the publication focuses on the likely culprits behind why some cancers are treatment‐resistant: cancer stem cells (CSCs). Authors employed multiple techniques, both in vitro on cells alone, to determine their invasiveness before administration and their tumorigenic capacity, and also in vivo on mice and chicken embryo’s CAM. Here, two variants were identified: adherent parental and those that formed mammospheres (CSCs). Furthermore, their interest was specified in FMCs expressing the CD133^+^ marker, as it was demonstrated that the remaining markers (CD24, CD44) were nonspecific for FMC CSCs. Additionally, the CSC’s sensitivity to radiation and chemotherapy was examined. Authors ultimately demonstrated that CD133^+^ cells exhibited a better ability to form spheres than CD133^-^ cells and were also more invasive. At the same time, mammospheres led to the appearance of larger tumors. However, no significant differences were found between the structures of the two tumor cell lines. Regarding their resistance to the applied therapies, in both cases (radiation and chemotherapy), they were very resistant compared to the adherent parental cells. It was emphasized that isolated CSCs exhibit characteristics of a mesenchymal phenotype, inducing potential metastasis. This observation was demonstrated by performing studies on CAM, where the ability of cells to invade the capillary system was observed. However, further studies will be needed to clearly confirm whether CSCs are capable of initiating tumorigenesis at distant sites.

Ademi et al. [34] described a relatively broad range of studies, as the authors examined a range of cancer cell lines, both human and animal, and demonstrated that not all lines are capable of tumorigenesis on CAM. However, those that proved capable (17CM98, K9STS, D‐17, and HAS—canine oral melanoma, soft tissue sarcoma, adherent osteosarcoma, and haemangiosarcoma, respectively) were selected, and further studies were conducted. In addition to assessing the established tumors, the angiogenesis process through real‐time observations of blood perfusion in vessels in close proximity to the tumor was investigated. This was done in response to two antiangiogenic monotherapies (Avastin; AVA and AZD3965; AZD) and combination therapy (AVA + AZD). The findings were as follows: AVA monotherapy significantly reduced growth in 17CM98 tumors and to a slightly lesser extent in D‐17. However, in each case, the AVA + AZD combination demonstrated the potential to shrink the primary tumor mass. Unfortunately, they were ineffective in limiting perfusion around the tumor. Regarding cell dissemination, AVA promoted invasion and expansion of the tumor, while AZD alone and AVA + AZD reduced these trends, particularly for the D‐17 line. It was also found no response to treatment in terms of tumor tissue hypoxia [34].

In contrast, studies conducted by Małek et al. [32] on two canine osteosarcoma lines of varying malignancy (OSCA‐8 and OSCA‐32) focusing on the extravasation process. Therefore, preliminary analyses of the accumulation of prepared nanocarriers and their overall cytotoxicity were performed, and then intravenously administered fluorescently labeled cells from both cell lines into the vascularized CAM. The authors observed the spread of cells from the bloodstream to adjacent places using fluorescence microscopy. Based on this, a conclusion was made that the previously stated notion that cell malignancy determines predisposition to metastasis is not entirely true. Because extravasation is one of the steps in the metastasis process, it was demonstrated no significant differences between the two cell lines. It should be noted that one cell line (OSCA‐8) was more aggressive than the other (OSCA‐32). Using cells treated with gold nanoparticles (AuNPs), the authors demonstrated that these nanocarriers had a positive effect on reducing the extravasation process, highlighting their potential antimetastatic properties.

## 5. Advantages and Disadvantages of the CAM Model

As outlined above, the CAM model is increasingly used to broaden its areas of application. It has unquestionable advantages, such as cost‐effectiveness compared to studies conducted on higher‐level organisms, rapid development, in just a few days, ease of handling (in ovo surpasses ex ovo), and good tissue availability (ex ovo surpasses in ovo). Nevertheless, its rapid daily development might be a disadvantage, as it does not provide enough time for reliable measurements. Moreover, studies permitted without the approval of the bioethics committee are characterized by a significant time limit, due to the well‐being and painlessness of the organisms subjected to the experiment. The aforementioned short cultivation time can be both an advantage and a disadvantage. First, in the case of the CAM model, the cells are not administered subcutaneously, as in rodents, requiring the use of a suitable gel matrix. Furthermore, tumor vascularization may depend on the tumor type, which, in the case of drug carrier studies, may prevent their accumulation despite the tissue availability for free drug through simple diffusion. Therefore, in some cases, testing the accumulation rate or activity of these carriers may be closely linked to the rapid vascularization of tumors and be limited to those that develop functional vessels in just 3–4 days. However, due to the small diameter of blood vessels, intravenous administration of drugs or formulations is very difficult, at least much more so than in rodent models. The size of tumors also differs between the types of cells used; however, they are small, of the order of 0.69 cm^3^ [38], diameter of about 3 mm [31] to 1 cm [39]. For this reason, the results obtained in the CAM model may be different from those obtained in mice. Importantly, the culture temperature of chicken embryos (37.5–38°C) is similar to mouse body temperature, allowing for direct comparison. However, human tumor tissue temperatures are typically higher than those of human bodies (38.5–39.5°C), given the smaller differences in body temperature between dogs and cats, indicating that the CAM model would be more appropriate for studies of cats and dogs than humans. Another aspect would be the role of the yolk sac, especially in toxicity studies of the drug and even drug carriers. It is unclear whether it serves as a biological sink for drugs, and some of the administered lipophilic substances are deposited there, reducing the actual drug toxicity, which would be higher for the rodent model. This is an open question that requires further investigation [40]. For this reason, some tests may not provide a complete picture, because they require more time needed for tissue to develop or to determine the presence of certain elements, such as immune system cells. However, its significant advantage is transparency and ease of visualization and observation of changes, and its circulatory system provides efficient and suitable conditions for the maintenance and development of all types of grafts—cells, tissues, or biomaterials. This would also not be possible if not for the natural immunodeficiency of CAM; otherwise, all xenobiotic grafts would be attacked by a properly functioning immune system, ultimately leading to rejection and death of the tested tissue. As stated by Ribatti [41], the chicken embryo is protected by a dual immune system based on the presence of B‐ and T‐cells. However, the chicken immune system only begins to function at around 2 weeks, specifically the first T‐ and B‐cells are detected on days 11 and 12, respectively. The chicken embryo only becomes immunocompetent by day 18. This aspect, along with the time‐limited nature of the research due to ethical committees, can pose a serious problem for studies investigating the immune system and its response. As pointed out by Garcia et al. [42], late‐stage chicken embryos develop only partial immunity because some abilities are reserved for the period after hatching. Perhaps one of the most key disadvantages and limitations of the CAM model may be also the fact that when compared to other generally used research models, due to its origin (avian), implicates differences with the metabolism, and physiology of mammals, this may suggest limited availability of antibodies, cytokines, antibiotics or other reagents needed to conduct a reliable study [1]. Most, if not all, commercially available cytokines, antibodies, and other reagents were designed for the human and mouse species. The CAM model, while similar in composition to mammalian immune systems (including dogs and cats), exhibits certain differences in the richness of immune cells due to its avian origin. As reported by Garcia et al. [42], the extensive repertoire of cytokines and chemokines found in humans has been confirmed to be present in chickens, with some exceptions. The authors cited the interleukin‐1 (IL‐1) family as an example, which consists of 11 cytokines in humans, but only four have been found in chickens to date, sharing the same biological functions. It has been found that avian cytokines share only 25%–35% amino acid sequence identity with their mammalian orthologs. Another advantage, but at the same time a disadvantage, may be sterility and resistance to negative conditions—this is the domain of in ovo culture because the shell provides safe and sterile conditions for the growth of the embryo and thus CAM development. Therefore, any interference, such as that related to ex ovo, reduces the possibility of counteracting infections [11]. A comparison of the advantages and disadvantages of the CAM as a model is shown in Table 3.

**Table 3:** Overview of positive and negative aspects of the CAM model.

Advantages	Disadvantages
Cost‐effective	Physiological differences
Ease of handling (in ovo)	Time limitation for conducting experiments
Rapid development	Risk of nonspecific inflammatory responses
Tissue availability (ex ovo)	Sterility (ex ovo)
Ease of visualization	Uneven tumor vascularization time
Natural immunodeficiency	Egg yolk as a drug sink
Ethically legal	Absent immune contribution

## 6. Future Perspectives of the CAM Model in Veterinary Diagnostics

The aforementioned achievements demonstrate the real potential of the CAM model in cancer research affecting both humans and animals. Although published researches have focused primarily on experimental studies, there are indications that the CAM model could be used in the future to aid in diagnostics in veterinary oncology. It has already been demonstrated that tumors can be cultured from both commercial cells [31, 34, 36] and those isolated directly from patient tissue [30], allowing for direct administration of patient material to these membranes. The model allows for the introduction of cells onto the surface and intravenously, thus enabling dual observation of the process of tumorigenesis, dynamics, angiogenesis, invasiveness, and metastasis. Thanks to the complexity and advancement of the CAM model compared to 2D (monolayered cell culture) and 3D (e.g., spheroids or organoids) cell culture models, it allows for understanding the ongoing processes and interactions between individual elements at a much higher level than is possible with cell line‐based studies. As it is well‐known, that the tumor microenvironment is not limited to cancer cells. It is a composition of normal and mutant host cells and supporting structures, such as vasculature and stromal components. Therefore, studies using cell lines represent only a fraction of what can be achieved with studies on complex organisms. One could speculate that this would be particularly beneficial when the nature of the pathological lesion visible on imaging (e.g., ultrasound) is unclear or completely unknown. Using techniques such as fluorescence or perfusion [32, 34] in vivo, as well as ex vivo using immunohistochemical staining, would allow for the characterization of the tumor under study in a relatively short time, determining its capabilities and limitations in a living organism. All this without the repetitive, stressful, and often invasive procedures performed on veterinary patients. As stated by Miebach et al. [37], the use of these techniques allows for the characterization of tumor tissue in terms of, among other things, apoptosis, the immune landscape, metastasis, invasiveness, and tumor growth. More specifically, through the use of transduced cells (often with luciferase) and the phenomenon of luminescence, it is possible to track cell migration and accumulation in embryonic tissues and organs. Moreover, by administering fluorescent dyes (e.g., fluorescein), it is possible to observe flow, vascular recruitment, and tumor perfusion. Staining, on the other hand, focuses on the precise characterization of excised tissue by targeting specific markers. Thanks to the development of the embryo and its vessels, the process of tumorigenesis can proceed much faster than in other models (e.g., mice) and is easier to visualize than in mammals (humans, dogs, and cats). The CAM is not limited to chicken embryos; research can be conducted on other avian organisms, including quail, turkeys, and ducks, which, as characterized by Kundeková et al. [1], have different capabilities (shorter or longer development time till hatching) and may predispose specific uses (research on angiogenesis, pathogenesis, and virulence, or environmental toxicity). These are not the only possible applications of the model. Dhayer et al. [43] have proven that the CAM model can be successfully used to evaluate the biocompatibility of all types of scaffolds thanks to its rich vascularization and the observation of inflammatory processes in response to the tested material. Furthermore, it allows for the observation and evaluation of material‐membrane integration. In this regard, it was demonstrated that the structure and properties of the CAM membrane also allow for the maintenance and development of transplanted chicken and mouse limb buds [44]. This makes the model undoubtedly suitable for advancing tissue engineering and regenerative medicine. It could also serve as a some sort of bioreactor for the propagation of material needed for molecular diagnostics. Such a use would be worthwhile when a sample collected from a veterinary patient is insufficient in size or cell quantity for further testing. Since Zabielska et al. [30] have proven that it is possible to isolate and successfully culture a tumor derived from a veterinary patient, and to treat the grown tumors with both available and innovative therapies, as described by [32, 34]. Hence, it means that the model is also suitable for testing response to various therapies, and the available options could be tested on patient tissues, thus tailoring treatment to the individual case. It is worth noting that the described applications remain purely speculative at the moment, as there are no sources describing its use in this way. Hence, the CAM model could bridge the gap between in vitro assays and in vivo mammalian models in veterinary oncology.

## 7. Conclusions

As described above, the CAM model can be considered an innovative tool for veterinary oncology. Its undisputed advantages include the ability to conduct research without the use of laboratory animals. Furthermore, it can also provide valuable information regarding the possibility of diagnosing the biological aggressiveness of the tumor. The extensive analyses conducted using numerous commercial and self‐isolated cell lines of various canine and feline cancers suggest that the use of CAM to a similar or identical extent as in human medical research is possible. Both successes and failures only underscore the validity of this statement, as there is never a perfect model for conducting research. We hope that the wider use of this model will soon contribute to a significant acceleration of the development and characterization of effective methods of treating various types of cancers in animals, not only domestic.

**Nomenclature**
17CM98: Canine oral melanoma cell line
3R: Replacement, reduction, refinement rule
AuNP: Gold nanoparticles
AVA: Avastin
AZD: Antiangiogenic monotherapy
BxPC‐3: Human pancreatic adenocarcinoma cell line
CAM: Chorioallantoic membrane
CD 133^+/−^, 24, 44: Cluster of differentiation 133^+/−^, 24, 44
CSCs: Cancer stem cells
D‐17: Canine adherent osteosarcoma cell line
DMEM: Dulbecco’s modified Eagle medium
EARA: European Animal Research Association
EMEM: Eagle’s minimal essential medium
FBS: Fetal bovine serum
FeLV: Feline leukemia virus
FFS1: Feline fibrosarcoma cell line 1
FFS3: Feline fibrosarcoma cell line 3
FMC: Feline mammary carcinoma cell line
FVAF1: Feline vaccine‐associated fibrosarcoma cell line
GLOBOCAN: Global Cancer Observatory
H&E: Hematoxylin and eosin
HAS: Canine oral melanoma cell line
HEPES: 4‐(2‐Hydroxyethyl)‐1‐piperazineethanesulfonic acid
IARC: International Agency for Research on Cancer
IHS: Immunohistochemical staining
IL‐1: Interleukin 1
K9STS: Canine oral melanoma cell line
Ki‐67: Marker of proliferation Ki‐67
NEAA: Nonessential amino acids
OSCA‐32: Canine osteosarcoma cell line
OSCA‐8: Canine osteosarcoma cell line
PCNA: Proliferating cell nuclear antigen
VAFS: Vaccine‐associated feline sarcoma.

## Disclosure

All authors have read and agreed to the published version of the manuscript.

## Conflicts of Interest

The authors declare no conflicts of interest.

## Author Contributions

**Anna Sczasny**: investigation, writing – original draft, writing – review and editing, visualization. **Jerzy Gubernator**: writing – review and editing. **Anna Jaromin**: conceptualization, writing – review and editing, supervision, project administration, funding acquisition.

## Funding

The publication of this article was supported by the Excellence Initiative – Research University Program for the University of Wrocław.

## Supporting Information

Additional supporting information can be found online in the Supporting Information section.

## Supporting information


**Supporting Information 1** Table S1: Most common tumor types affecting dogs—their incidence and factors resulting in increased risk [18, 20].

**Supporting Information 2** Table S2: Most common tumor types affecting cats—their incidence and factors resulting in increased risk [45, 46].

## Data Availability

All data described in this review were obtained from the publications listed in the References section.
