# Supplementary material for: Innovative Chorioallantoic Membrane Model as Valuable Tool in Diagnostics and Testing of Domestic Animal Cancers
Source: Transbound Emerg Dis. 2026 Jan 3;2026:1876572. doi: 10.1155/tbed/1876572 (PMC12764293; doi:10.1155/tbed/1876572)
Supplement: Supplementary file 1 — Supporting Information 1 Table S1: Most common tumor types affecting dogs—their incidence and factors resulting in increased risk [18, 20]. [file TBED-2026-1876572-s002.docx]

Table S1. Most common tumor types affecting dogs - their incidence and factors resulting in increased risk [18], [20].

| Cancer Type | Incidence (%) | Increased Risk Factors | | |
| --- | --- | --- | --- | --- |
|  |  | Breed | Age | Neutering Status |
| Mast Cell Tumor | 9.97 | Swiss Mountain Dog, Retriever, Buldog, Boxer | 3-9 years | Neutered male and female dogs |
| Adenoma/Adenocarcinoma | 7.54 | Yorkshire Terrier, Collie, Poodle, Cocker Spaniel | >2.5 years | Neutered male dogs |
| Melanocytic Tumor | 5.26 | Rottweiler, Setter, Poodle, Doberman, Cocker Spaniel | >6 years | Neutered female dogs |
| Squamous Cell Carcinoma | 4.92 | Schnauzer, Poodle, Cocker Spaniel | >3 years | Comparable |
| Lymphoma | 4.70 | West Highland White Terrier, Swiss Mountain Dog, Rottweiler, Boxer | >3.5 years | Neutered male and female dogs |
| Fibroma/Fibrosarcoma | 3.72 | Setter, Shepherd, Rottweiler, Collie, Great Dane | >3 years | Comparable |
| Haemangioma/Haemangiosarcoma | 3.22 | Boxer, Shepherd, Swiss Mountain Dog | >4 years | Neutered female dogs |
| Osteoma/Osteosarcoma | 0.99 | Rottweiler,  Great Dane, Doberman | >5 years | Neutered male dogs |
| 'Melanocytic Tumor' by means of 'Melanocytomas and Melanomas'; 'Haemangioma/Haemangiosarcoma' by means of 'Soft Tissue Tumors and Sarcomas'; 'Osteoma/Osteosarcoma' by means of 'Osseous and chondromatous neoplasms'; 'Comparable' refers to similar risk for both sex | | | | |

Table S2. Most common tumor types affecting cats - their incidence and factors resulting in increased risk [45], [46].

| Cancer Type | Incidence (%) | Increased Risk Factors | | |
| --- | --- | --- | --- | --- |
|  |  | Breed | Age | Neutering Status |
| Adenoma/Adenocarcinoma | 19.1 | Siamese, Oriental Shorthair, Chartreux | >2 years | Neutered male and non-neutered female cats |
| Fibroma/Fibrosarcoma | 18.4 | Mixed Breed | >7 years | Neutered female cats |
| Lymphoma | 15.6 | Somall, Oriental Shorthair, Siamese | <5 years | Neutered male, female and non-neutered male cats |
| Squamous Cell Carcinoma | 9.9 | European Shorthair | >2 years | Neutered male, female and non-neutered female cats |
| Osteoma/Osteosarcoma | 0.05 | No Specific | >8 years | No Specific |
| 'No Specific' refers to lack of prevalent representative | | | | |
