# Supplementary material for: Innovative Chorioallantoic Membrane Model as Valuable Tool in Diagnostics and Testing of Domestic Animal Cancers
Source: Transbound Emerg Dis. 2026 Jan 3;2026:1876572. doi: 10.1155/tbed/1876572 (PMC12764293; doi:10.1155/tbed/1876572)
Supplement: Supplementary file 2 — Supporting Information 2 Table S2: Most common tumor types affecting cats—their incidence and factors resulting in increased risk [45, 46]. [file TBED-2026-1876572-s001.docx]

Table S2. Most common tumor types affecting cats - their incidence and factors resulting in increased risk [45], [46].

| Cancer Type | Incidence (%) | Increased Risk Factors | | |
| --- | --- | --- | --- | --- |
|  |  | Breed | Age | Neutering Status |
| Adenoma/Adenocarcinoma | 19.1 | Siamese, Oriental Shorthair, Chartreux | >2 years | Neutered male and non-neutered female cats |
| Fibroma/Fibrosarcoma | 18.4 | Mixed Breed | >7 years | Neutered female cats |
| Lymphoma | 15.6 | Somall, Oriental Shorthair, Siamese | <5 years | Neutered male, female and non-neutered male cats |
| Squamous Cell Carcinoma | 9.9 | European Shorthair | >2 years | Neutered male, female and non-neutered female cats |
| Osteoma/Osteosarcoma | 0.05 | No Specific | >8 years | No Specific |
| 'No Specific' refers to lack of prevalent representative | | | | |
